# Fellow-Workers and the Roll of Honour

**Published:** 1914-10-03

**Authors:** 


### ROUND THE WORLD.

[The Editor Invites Early Information and Contributions Marked "Fellow-Workers."]

### Fellow-Workers and the Roll of Honour.

Among the Cambridge men associated with the 1st Eastern General Hospital there is Dr. John Aldren Wright, assistant physician and pathologist to Addenbrooke's Hospital. A student of St. Mary's Hospital, as well as of Cambridge University, he qualified M.B., B.C. Cantab. in 1904, and became an M.R.C.P. Lond. three years ago. His writings on professional subjects include several observations on brain lesions.

The following practitioners form the staff of captains at the 1st Eastern General Hospital: Mr. E. J. Y. Brash, Dr. H. H. Brown, Mr. S. W. Curl, Dr. J. F. Gaskell, Mr. J. C. W. Graham, Dr. G. S. Haynes, Dr. W. Malden, Mr. D. Mallam, Dr. B. H. Nicholson, and Dr. G. F. Rogers.

Dr. W. H. Symons, the medical officer of health for Bath, has offered himself for service abroad if required. He is a major in the R.A.M.C., Territorial Force, and an old Volunteer.

Dr. Robert Arthur Milligan is a Northampton practitioner, and holds the post of surgeon to the General Hospital there. Qualifying M.R.C.S. Eng. from Guy's Hospital in 1881, he became an M.D. Durh. after he had been in practice many years. Before settling in Northampton he held the post of resident obstetric officer to Guy's Hospital, resident clinical assistant at Bethlehem Royal Hospital, and anæsthetist to the Evelina Hospital for Sick Children.

The 2nd Eastern General Hospital of the Territorial establishment centred in the new Brighton Grammar School, is staffed by many well-known practitioners living in the Brighton district, of whom Dr. E. F. Maynard and Mr. F. J. Paley were called out with the rank of lieutenant-colonel on first mobilisation, together with Mr. A. H. Buck, Mr. W. A. Bowring, Dr. D. G. Hall, and Dr. C. F. Bailey as majors. Also the following gentlemen, who are now serving as surgeon-captains: Dr. A. G. Bate, Dr. W. H. Brailey, Dr. A. M. Colcutt, Dr. A. M. Daldy, Dr. F. A. S. Hutchinson, Dr. H. Gervis, Mr. C. N. Chadborn, Dr. J. Shardlow, Dr. L. A. Parry, Mr. H. H. Taylor, Mr. W. R. Wood, and Dr. R. Whittington.

Mr. Edward Forster Maynard has for some years been surgeon to the Sussex County Hospital, and is also honorary medical officer to the St. Bernard's Home for Invalid Gentlewomen at Hove, as well as honorary medical referee to the St. John's Home of Rest at Mentone. An Edinburgh and Guy's student, he qualified as graduate of his University in 1889, subsequently becoming an M.D. Ed., and M.R.C.P. Lond.

Mr. Arthur Herbert Buck, F.R.C.S. Ed., is a "Bart.'s" man who holds a prominent position on the staff of the Sussex County Hospital, where soon after qualification in 1892 he held the post of house-surgeon. Mr. Buck won the Brackenbury Scholarship in surgery at St. Bartholomew's, and at one time was clinical assistant in the throat department and house-surgeon there. His present appointments include those of surgeon to the Brighton, Hove, and Sussex Throat and Ear Hospital, and consulting surgeon to the Brighton Dental Hospital.

Mr. Frederick John Paley, M.D., Lond., is a "Bart.'s" man who qualified M.R.C.S. Eng., L.R.C.P. Lond., and M.B. Lond., and subsequently settling in Brighton became associated with public institutions there, and, notably, surgeon to the Sussex County Hospital. Formerly he was honorary surgeon to the Brighton and Hove Lying-in Institution.

Mr. Walter Andrew Bowring, F.R.C.S. Eng., is one of the assistant surgeons at the Sussex County Hospital, and also holds the post of surgeon to the Brighton and Hove Hospital for Women. Studying medicine at St. Thomas's Hospital, he qualified in 1892, and subsequently became senior obstetric house-physician there and also senior resident medical officer to the Royal Free Hospital. In early years he was one of the prosectors at the Royal College of Surgeons.

Dr. Charles Frederick Bailey, M.D. Lond., M.R.C.P. Lond., D.P.H., is a Brighton man who has devoted a good deal of time to electro-therapeutics. Qualifying from St. Bartholomew's in 1882, he worked for some time in North London, and, eventually settling in Brighton, became a member of the staff of the Sussex County Hospital, where he is now assistant physician and medical officer in charge of the electrical department. He is the author of many communications on the medicinal value of *x*-rays and various forms of electricity.

Dr. Donald George Hall, M.D. Cantab., M.R.C.P. Lond., is assistant physician to the Royal Sussex County Hospital, physician to the St. Bernard's Home for Invalid Gentlewomen at Hove, and physician to the St. John's Home for Children at Brighton. As a student of Cambridge and Edinburgh, Dr. Hall had a distinguished academic career, taking first-class honours in the Natural Science Tripos at the former University in 1897.

Liverpool practitioners are largely interested in the 1st Western General Hospital which has been established in connection with the Fazakerley Hospital, where Sir James Barr, Sir Ronald Ross, Mr. R. Parker, and Mr. W. Alexander are lieutenant-colonels; the following gentlemen being majors in this unit: Dr. T. R. Bradshaw, Dr. T. Bushby, Dr. P. Davidson, Mr. R. Jones, Mr. R. W. Murray, Mr. F. T. Paul, Mr. J. Utting, and Mr. E. A. Browne.

Sir Ronald Ross, who was created a K.C.B. three years ago for his splendid efforts in the advancement of tropical medicine, is professor of that subject at Liverpool University and in the Liverpool School of Tropical Medicine. A student of St. Bartholomew's Hospital, he entered the Indian Medical Service in 1881, and some ten years later began specially to study malaria, ultimately undertaking with success the experimental verification of the mosquito theory of that disease. He retired from the Indian Medical Service with the rank of major in 1899, and three years later was awarded the Nobel prize for Medicine. Sir Ronald Ross is an M.D., F.R.C.S., and F.R.S., in addition to which he has been the recipient of various honorary degrees.

